# Tissue and sperm handling before assisted reproductive technology (ART): A systematic review

**DOI:** 10.1080/2090598X.2021.1954801

**Published:** 2021-07-22

**Authors:** Rafael Favero Ambar, Marcello M. Gava, Milton Ghirelli-Filho, Ivan H. Yoshida, Thais Serzedello De Paula, Sidney Glina

**Affiliations:** a Urology Department at Centro Universitario Em Saude Do ABC – FMABC, Santo André, Brazil; b Ideia Fertil Institute of Human Reproduction, Santo André, Brazil; c Crio Brasil/Fairfax Cryobank Brasil, São Paulo, Brazil

**Keywords:** Male infertility, sperm selection, assisted reproduction, cryopreservation, azoospermia, sperm retrieval

## Abstract

**Objective:**

: To explore the medical literature on techniques of tissue and sperm handling after surgical retrieval for intracytoplasmic sperm injection (ICSI).

**Methods:**

: A search was performed in PubMed and Google Scholar databases, according to a modified Preferred Reporting Items for Systemic Reviews and Meta-Analyses (PRISMA) guideline, considering the studies investigating tissue handling and sperm selection techniques for ICSI.

**Results:**

: Overall, 42 articles were included in this study, investigating sample handling, methods for sperm selection, and the use of chemical compounds to improve sperm motility and fertilisation rates.

**Conclusion:**

: The ideal sperm handling method should provide a high sperm count, high vitality and appropriate sperm function, without side-effects. In this review the most common and useful techniques are described and the best combination strategies discussed in clinical scenarios.

## Introduction

Rapid advances have occurred in recent decades with respect to male infertility, since the introduction of intracytoplasmic sperm injection (ICSI) in 1992, in the treatment of severe male factor infertility [1]. Male factor infertility is present in almost half of couples who fail to conceive after a year of attempts. Azoospermia, defined as absence of sperm in ejaculate after centrifugation, affects 10–15% of infertile men. It may occur due to impaired sperm production, the most severe form of male-factor infertility, named non-obstructive azoospermia (NOA), or due to obstruction of the male reproductive tract, obstructive azoospermia (OA) [2].

In 1994, the first series of ICSI using sperm retrieved from the epididymis was reported in a group of men with OA [3]. In the following year testicular sperm retrieval using an open biopsy (testicular sperm extraction [TESE]) was performed in 15 men with NOA [4]. As result, the development of different methods of sperm recovery allowed patients with OA and NOA considered sterile to father their biological children.

Procedures for sperm retrieval may be coordinated with oocyte retrieval so that fresh sperm can be used for ICSI. In contrast, other centres offer sperm retrieval with cryopreservation with the intention of using thawed sperm at a future date [5]. Outcomes for the use of fresh vs frozen sperm for assisted reproductive technology (ART) in men with NOA have been compared. A meta-analysis compiled data from 11 studies comprising 574 ICSI cycles and reported no difference between fresh and frozen sperm in clinical pregnancy rate or fertilisation rate [6].

Today, many clinicians are increasingly inclined to perform ICSI with testicular sperm in non-azoospermic patients who failed implantation and have high levels of DNA damage [7], but it is still controversial, and the decision should be made on a case-by-case basis [8]. Several sperm selection techniques have been proposed to improve the chance of recovering surgically retrieved mature sperm with high DNA integrity and preserved structure [9].

The challenge with sperm retrieval in NOA is that testicular sperm are often non-motile, extremely rare and a spermatogenesis focus should be found. Therefore, sperm extraction from patients with NOA requires special care and techniques of the embryology team [10]. However, spermatogenesis remains preserved in patients with OA, so sperm retrieval has more successful retrieval rates and less complex sperm handling than NOA.

In the present article, we provide a brief literature review on surgical sperm retrieval techniques (SRT), as well as a systematic review on sample handling and sperm selection for ICSI. Finally, clinical cases in which these techniques were performed are reported in a practice-based approach.

## Methods

A literature search was conducted on PubMed and Google Scholar databases looking for studies investigating tissue handling and sperm selection techniques for ICSI. The search strategy according to a modified Preferred Reporting Items for Systemic Reviews and Meta-Analyses (PRISMA) guideline included the following terms: ‘sperm selection’, ‘sperm separation’, ‘sperm processing’, ‘sperm injection, intracytoplasmic [MeSH Terms]’, ‘assisted reproduction technology’, ‘assisted reproduction techniques’. Studies performed with surgically retrieved human sperm, published in English were included. Manual search of references of the selected studies was also performed.

## Results

**Figure 1.**
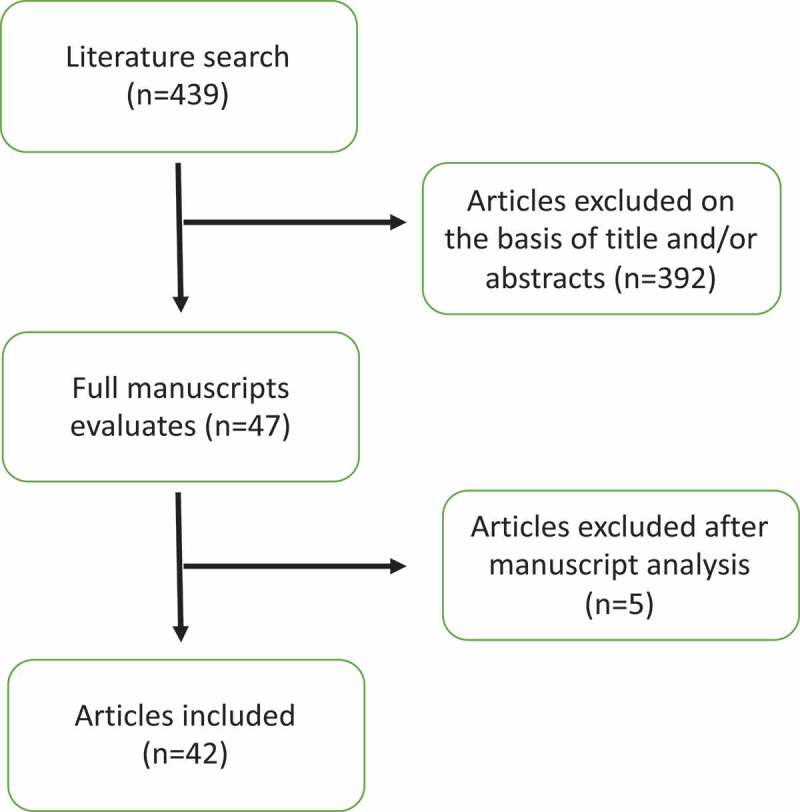
PRISMA flowchart of literature search

The search was performed as previously described and yielded 439 articles. After careful analysis of title and abstracts, 392 were excluded. The 47 remaining articles were screened, and 42 met the inclusion criteria (Figure 1).

Among the included studies, seven concern sample handling [11–17], 10 articles investigated the use of chemical compounds to improve sperm motility, such as pentoxifylline and theophylline [18–27]. The use of calcium ionophore was analysed in four studies [28–31]. The identification of primordial cells in the sample was reported in three studies [32–34]. Methods of sperm selection were investigated in 12 articles [35–46]. Finally, different techniques of sample handling for sperm freezing/thawing were discussed in six studies [47–52].

## Sperm retrieval techniques (SRT)

Sperm retrieval techniques are surgical methods to obtain spermatozoa from the testicle or epididymis to be used in ICSI [53]. The most important outcome when assessing sperm extraction is the sperm-retrieval rate (SRR). The SRR indicates the percentage of success in sperm recovery, which means that when sperm is found it is considered successful. The SRR is calculated by dividing the number of successful cases by the number of total cases performed. In OA, the SRR is near 100%, the ICSI outcomes do not differ if testicular or epididymal sperm is used [54].

Percutaneous epididymal sperm aspiration (PESA) and microsurgical epididymal sperm aspiration (MESA) are the most used methods to harvest epididymal sperm in cases of OA and testicular sperm aspiration (TESA) for testis.

Regardless of technique, in NOA, the SRR ranges from 30% to 63%, with a median rate of 50% [55]. Microdissection-TESE (micro-TESE) and TESE are the most used methods to harvest testicular sperm in cases of NOA. In 1999, micro-TESE was described estimating that the performance of a micro-TESE was 1.5-times more likely to retrieve sperm [56,57].

In selected cases, non-azoospermic patients who failed implantation and have high levels of DNA damage, sperm can also be retrieved from testis. In such cases, epididymal techniques should not be performed due to the risk of obstruction [8].

Indications, advantages and disadvantages of SRT are summarised in Table 1.

**Table 1.** Indications, advantages and disadvantages of SRT

Technique	Indications	Advantages	Disadvantages
PESA	OA	Fast and repeatable; no microsurgical expertise required; minimal morbidity and fast recovery; local anaesthesia and low cost	Few sperm retrieved; limited possibility for cryopreservation; risk of epididymal damage and haematoma
MESA	OA	Large number of sperm retrieved; reduced surgical complications; available cryopreservation with excellent results	Microsurgical expertise required; increased cost; general anaesthesia required; surgical microscope and microsurgical instruments required; postoperative discomfort
TESA	OA and NOA^1^	Fast and repeatable; no microsurgical expertise required; minimal or mild morbidity and fast recovery; local anaesthesia and low cost. Can be used as first choice in OA or in failed PESA	Few sperm retrieved; limited possibility for cryopreservation; risk of testicular atrophy and haematoma; low success rate in NOA
TESE	OA and NOA^1^	Fast and repeatable; no microsurgical expertise required	Low success rate; few sperm retrieved; limited possibility for cryopreservation; postoperative discomfort
Micro-TESE	NOA	Higher success rate; larger number of sperm retrieved	Microsurgical expertise required; increased cost and general anaesthesia; surgical microscope and microsurgical instruments required; postoperative discomfort

## Surgically retrieved sample handling

### Sample preparation

#### Dissection methods

Surgical sperm extraction techniques from the testis and epididymis are well established, and protocols for handling these samples may vary between laboratories. It is not suitable to use seminal processing techniques of ejaculated samples for these samples due to poor concentration or low motility of the retrieved sperm [11].

Epidydimal retrieved sperm generally need less laboratory manipulation, once surgeons use supplemented and heated culture medium in the procedure, and sperm is extracted directly from the epididymis, without the necessity of dissecting tissue, as other approaches [58].

Testicular sperm extraction and tissue processing require a gentle manipulation to acquire a good cell yield. The biopsied tissue is manually fragmented to release seminiferous tubules (STs) content. This is a crucial procedure, as it is the only method to recover sperm [11].

Different procedures can be applied to release sperm from the STs:

- scissors cutting
- scalpel tear
- 26-G needle dissection

In all the applied techniques above, the untied spermatozoa remain attached to STs, which often hinders sperm visualisation.

It is common sense that better results would be obtained if testis sperm extraction is performed the day before oocyte retrieval, to ensure an incubation of this sample in supplemented culture medium, which improves sperm motility [11,12,57–59].

Laboratory staff may also choose to use some of the contiguous techniques described below.

#### Erythrocyte lysis

Surgically recovered sperm samples usually present red blood cells, which may hinder sperm identification and uptake, especially if they are immotile. In such cases, the use of an erythrocyte lysis solution is a possible alternative [60].

After testicular tissue dissection or epidydimal aspiration, the sample should be centrifuged, the supernatant discarded and should be added ~1–2 mL of buffer solution. The amount to be used depends on the pellet size [16].

Buffer solution is composed of (in mM) NH_4_Cl (155), KHCO_3_ (10) and ethylenediamine tetra-acetic acid (EDTA, 2) at pH 7.2. This solution is responsible for erythrocyte lysis and does not affect sperm viability [16,60].

This mixture should be incubated at room temperature for 5 min. Then, heated and supplemented culture medium is added to remove the buffer solution, and the sample is centrifuged again, the supernatant discarded and pellet re-suspended in 0.5–1 mL of supplemented and heated culture medium. After this procedure, the sample must be evaluated under a microscope [16,60].

This procedure is not time-consuming and it does not have negative effects on fertilisation potential [13].

#### Enzymatic digestion

Surgical samples where sperm is not easily found require some specific procedures to ensure the recovery of these cells. Enzyme digestion is an alternative to improve sperm recovery [13,14].

In men with NOA, sperm production is low, and most of them are adhered to testicular tissue, mechanical extracting is time-consuming, and, if improperly performed, might damage sperm. Thus, performing enzymatic digestion of testicular tissue is an efficient tool to improve sperm recovery [60].

Baukloh [15] in 2002, carried out a multicentric and retrospective study comparing fertilisation rates and concluded that the use of immotile sperm and elongated sperm selected enzymatically presented a higher fertilisation rate than untreated ones.

The purpose of these enzymes is to digest the collagen that composes basement membrane and extracellular matrix, helping sperm release from tissue. Different enzyme varieties are used, such as collagenase types I and IV, and DNase I. The difference between them is the respectively used protocol [13,14,60].

Wöber et al. [17] managed to recover 7–26% of samples classified as having no sperm after microtubule dissection, being a viable alternative in poor prognosis cases.

#### Primordial cell identification

The use of nuclear-fast/picroindigocarmine staining (NF-PICS) may help in clinical management of azoospermic patients in a rapid and non-invasive form by identifying primordial germ cells in ejaculate or biopsied testicular tissue, indicating a possible spermatogenesis focus [32,34].

Azoospermia is defined as the complete absence of sperm from the ejaculate. For a precise diagnosis, it is necessary to centrifuge the entire semen sample and look for sperm in the pellet to ensure it is completely absent [61], but frequently the observer may have difficulty in applying this technique, because sperm may be present in a small quantity, and its morphology altered, which hinders its identification.

With NF-PICS staining the sperm head is red and tail green. The same staining pattern may be observed in primordial cells (spermatids and spermatogonia).

In case of TESE, primordial cells identification may be of great value because this finding indicates a possible spermatogenesis focus. Occasionally, the morphology of these cells may be altered due to the extraction method of microtubules, hence applying a differential and specific staining may help in identifying these cells.

Hendin et al. [34] carried out a study comparing histological findings of prognostic testicular biopsy and NF-PICS staining in centrifuged ejaculates. A positive correlation was observed between both analyses, concluding that the use of staining may avoid a surgical testicular biopsy procedure. NF-PICS can be easily included in andrology laboratories routines as an inexpensive and effective way to identify sperm in azoospermic patients [33,34].

### Sperm selection

#### Pentoxifylline

Pentoxifylline is a phosphodiesterase enzyme inhibitor, derived from methylxanthine. It inhibits cyclic adenosine monophosphate (cAMP) phosphodiesterase, increasing intracellular cAMP concentration and tyrosine phosphorylation at the sperm tail level [19]. cAMP plays a role in sperm kinematics and in the acrosome reaction. Accordingly, if intracellular cAMP concentration is increased, sperm motility also increases, as well as the acrosome reaction [20].

Pentoxifylline has become a tool to increase sperm motility and fertilisation capacity in asthenozoospermic samples or in selecting immotile testicular sperm; however, still with controversial results [21,22]

Some studies reported an improvement in fertilisation rate, and consequently, in the number of available embryos, but no benefit in clinical results [23,24]. Other authors have shown no difference in any of the evaluated parameters [25].

An important highlight is that the pentoxifylline concentration in an adopted protocol may affect laboratory results. Concentrations in the micromolar range did not have a negative influence on embryo development, but in millimolar, however, demonstrated deleterious effects, leading to developmental embryo block in early stages [26,27].

Although clinical evidence is still conflicting about this techniques benefits, adding pentoxifylline simplifies sperm selection by inducing motility, giving the advantage of significantly reducing the time-consuming identification of viable sperm [24].

#### Theophylline

There is still a small representation in literature of using theophylline in spermatic stimulation. This technique principle is similar to pentoxifylline, increasing intracellular cAMP.

A single, well-structured study was developed by Ebner et al. [18], in which 65 patients were selected and submitted to TESE for various causes. Those samples were frozen to perform ICSI in the near future. After thawing, samples were divided into two groups: in one, theophylline was added to sample and in the other not. The results showed that 98.5% of the samples with theophylline had improved seminal motility, as well as oocyte fertilisation and pregnancy rates.

#### Calcium ionophore

Calcium ionophore has the main function of stimulating the acrosome reaction, to improve oocyte fertilisation rates. The literature on this topic is scarce and the most well-structured studies are slightly old and present conflicting results. Studies such as those of Yovich et al. [28] and Ebner et al. [29], demonstrated the effectiveness of using a calcium ionophore in in vitro fertilisation parameters.

Other studies such as those of Parinaud et al. [30] and Liu et al. [31], showed no advantages in calcium ionophore use, or classified it with limited importance in clinical practice.

#### Hypo-osmotic swelling (HOS) test

The HOS test is used to define functional integrity of sperm plasma membrane and was initially introduced as a functional assay to diagnose infertility [35]. Sometimes spermatozoa may have their membrane physically intact, but functionally inactive. Therefore, HOS provides supplementary information about sperm function. Rossato et al. [36] demonstrated that the exposure of sperm to hypo-osmotic conditions leads to the influx of water into the cytoplasm, expanding the volume of sperm that stretches the sperm membrane.

The main indication to perform the HOS test, according to the WHO 2010 manual, is in total asthenozoospermia cases, aiming to choose viable sperm for ICSI [61,62].

The published data remains inconclusive when correlating values found in the HOS test with ICSI results. Some studies have shown benefits of using the HOS test for sperm selection for ICSI with better quality embryos and higher implantation rates [39–41].

Despite the potential benefits associated with the use of HOS, even sperm with a low degree of HOS have the same fertility potential when performing the ICSI method, and therefore, the use of HOS is questionable for ICSI cycles [42–44].

#### Sperm tail flexibility test

This test consists of evaluating the viability of immotile sperm in a sample by observing if they have their tail flexible. This test is done by moving the sperm tail up and down with an ICSI needle, without moving its head [37]. In cases where the head moves along with tail, the sperm is not considered feasible to be used.

The use of frozen-thawed testicular sperm selected by this technique was analysed and the results were the same when comparing to the use of motile sperm. Besides that, the live-birth rate was similar in both groups [38].

The main advantage of this technique is that it uses only mechanical touch and does not require any activating compounds that could compromise embryo development. In addition, ICSI can be performed immediately after this test. However, this analysis is not 100% accurate and requires a highly-qualified laboratory staff [63].

#### Laser-assisted immotile sperm selection (LAISS)

The laser has been widely used in ART and is currently present in most laboratories. Another application for this tool is in the identification of immotile, but viable sperm [45]. A single laser shot is performed at the tip of the flagellum, and if sperm is alive, its tail will become curled or waved.

The use of this technique was shown to be similar when compared to the HOS test [46]. However, the main advantage of LAISS is that it does not require chemical substances to induce motility and, therefore, we do not expect associated side-effects [63].

## Tissue and sperm freeze–thawing

### Different techniques

Sperm cryopreservation is an essential and well-established practice in ART. The slow-freezing method has been widely used in sperm cryopreservation, allowing to preserve a huge sample volume with acceptable results regarding vitality and motility recovery after thawing [51].

Slow freezing stages consist of temperature decreasing, cell dehydration and storage [47].

Cryoprotectant agents are used to minimise cryoinjury, preventing intracellular ice formation. They control the water relocation from intracellular to extracellular environment [47,51].

The ideal cryoprotectant should have easy cell penetration, low toxicity and be soluble in water [51,52]. There are different cryoprotectants with their respective formulations and protocols available.

In freezing and thawing procedures, the cell membrane undergoes an important structural change because lipids freeze faster than membrane proteins. Besides that, there is also an alteration in hydrocarbons arrangement, which decrease their fluidity and, consequently, alters membrane permeability. These facts are due to the transition from membrane liquid phase to gelatinous state of cryopreserved cell [51,52].

It is necessary to control freezing velocity and add a cryoprotectant to assist cell survival, minimising then the occurrence of any sort of irreversible damage in the cell during this transition [51,52].

According to the WHO (2010), ~20–70% of sperm may not survive or suffer any irreversible damage during freezing/thawing processes. This wide variation is due to seminal sample quality before freezing [61].

It is known that sperm extracted from the epididymis or testis generally have poor concentration and low motility, so there was a necessity to develop a different method to freeze these samples [48].

In 2004, Isachenko et al. [49] developed sperm vitrification. Its success is based on two prime aspects: the very fast cooling before liquid nitrogen storage and the ultra-fast heating. These steps prevent intracellular ice formation, which is potentially lethal to the cells. Likewise, very fast heating step avoids re-crystallisation.

However, this technique allows the freezing of a very small volume of cells, as during devitrification intracellular ice formation and a mechanical damage (cracks) in the vitrified cell matrix may occur, which is potentially lethal to the cells [49,64].

Many sperm vitrification protocols were developed without using cryoprotectants, mostly with the use of sucrose and a low count of sperm. The main difference in protocols is based on different types of storage, through commercially devices available: cryoloop, drops (microdroplet), cryotop, cell sleepers or spermVD [64].

The vitrification technique is used for the storage of a few selected sperm through a micromanipulator, being especially favourable in surgical retrieval obtained sperm. Sperm vitrification may be an alternative to traditional freezing, with better results in sperm survival rate and DNA damage [47]. Le et al. [50] (2019) also highlighted a better morphology in spermatozoa after vitrification when compared to traditional freezing.

### Cryopreservation of testicular tissue

Paediatric cancer treatments have improved significantly in recent years, reaching an aggregate 5-year survival rates of >80%, and the majority of patients will live into adulthood [65]. On this basis, increased interest has risen about strategies for fertility preservation in children and pre-pubertal boys who have malignant diseases.

Testicular tissue cryopreservation can be safely obtained by performing a wedge biopsy before starting gonadotoxic treatments [66]. However, at this point it remains an experimental option, as no pregnancy from cryopreserved testicular tissue has been achieved to date [66,67].

## Clinical cases

As discussed above, there is wide variability of sperm selection methods, and each andrology laboratory may use different strategies to better handle sperm and testicular tissue before ICSI. In order to provide a practical and useful understanding of the techniques discussed, we present in this section two clinical cases of couples with severe male factor infertility undergoing ICSI.

### Case 1

A 25-year-old male, seeking medical assistance after identifying testicular lumps on self-examination was diagnosed with a testicular tumour. Concerned about fertility preservation, the physician ordered a semen analysis, which showed azoospermia. The patient was referred to a male infertility centre and after shared decision-making, it was decided to proceed with onco-TESE. As soon as the testicular tissue was obtained, we proceeded with shredding and fine mincing of the tissue with rupture of the STs. The suspension was assessed under an inverted microscope and no sperm was identified. It was decided to proceed with incubation of the pelleted testicular suspension with collagenase IV for 1 h. The digested solution was centrifuged, and supernatant cell suspension was washed with buffered medium, and the pellet was assessed by droplets under oil and rare sperm were identified. The formed pellet was placed into heated culture medium micro-drops in a Petri dish, covered by oil, and in the same plate we placed SpermVD (Sperm VD assisted reproduction device, developed by MFC global) device to perform sperm vitrification, as described by Berkovitz et al. [68].

Due to the very low count of sperm, vitrification was the technique of choice for cryopreservation. Approximately 10 spermatozoa were placed in each well of the device with the aid of an ICSI needle using an inverted microscope.

After orchidectomy, this patient underwent chemotherapy and follow-up evaluations showed that the treatment was successful. After 5 years, the patient and his partner presented to the clinic again, seeking ART. After complete evaluation of the couple, it was decided to perform ICSI using the cryopreserved sperm, as semen analysis showed azoospermia. The frozen sample was thawed by removing the device from the liquid nitrogen and placing it in a Petri dish covered with heated oil. The sperm were removed from the SpermVD with the aid of an ICSI needle and the oocyte fertilisation procedure was performed.

### Case 2

A 32-year-old male was admitted for fertility evaluation, reporting failure to conceive after 2 years of regular intercourse. He had an unremarkable past medical and surgical history. On physical examination, both testes were normally descended with decreased size and consistency. No other abnormalities were identified. His spouse was 26-years-old and had no gynaecological problems. Two semen analysis confirmed azoospermia after centrifugation. Blood tests revealed normal levels of testosterone, LH, prolactin, oestradiol and high FSH, karyotype 46 XY and no Yq microdeletion was found.

The couple was counselled about treatment options and decided to proceed with diagnostic TESA before ART. After TESA sample processing no sperm was identified; however, when NF-PICS was applied, primordial germ cells were stained. The pathology report result was Sertoli cell-only syndrome. Nonetheless, in light of the NF-PICS finding, it was decided to proceed with an attempt of sperm retrieval by micro-TESE along with ovarian stimulation for ICSI, on the day before oocyte aspiration. Concerning the micro-TESE sample, immotile sperm were identified after careful shredding and mincing of the tissue. Due to immobility, pentoxifylline was used to increase motility, ensuring the use of viable motile sperm for ICSI.

## Conclusion

The basic principle of ART is to combine gametes in order to achieve higher fertilisation, pregnancy and live-birth rates. Aiming for better clinical results, the interest on sperm has increased in recent years.

The ideal sperm handling method should provide a high sperm count, high vitality and appropriate sperm function, without side-effects. After decades of experience, the best strategy of testicular tissue and sperm handling for ART has not been completely defined. In the present review the most common and useful techniques have been described and the best combination strategy should be identified for each case.

## Abbreviations

ART: assisted reproductive technology; cAMP: cyclic adenosine monophosphate; HOS: hypo-osmotic swelling; ICSI: intracytoplasmic sperm injection; LAISS, laser-assisted immotile sperm selection; MESA: microsurgical epididymal sperm aspiration; NF-PICS: nuclear-fast/picroindigocarmine staining; NOA: non-obstructive azoospermia; OA: obstructive azoospermia; PESA: percutaneous epididymal sperm aspiration; PRISMA: Preferred Reporting Items for Systemic Reviews and Meta-Analyses; SRR: sperm-retrieval rate; SRT: sperm retrieval techniques; STs: seminiferous tubules: TESA: testicular sperm aspiration; (micro-)TESE: (microdissection-) testicular sperm extraction.

## References

[1] Palermo G, Joris H, Devroey P, et al. Pregnancies after intracytoplasmic injection of single spermatozoon into an oocyte. Lancet. 1992;340:17–18. 1351601 10.1016/0140-6736(92)92425-f

[2] Agarwal A, Mulgund A, Hamada A, et al. A unique view on male infertility around the globe. Reprod Biol Endocrinol. 2015;13:37. 25928197 10.1186/s12958-015-0032-1 PMC4424520

[3] Tournaye H, Devroey P, Liu J, et al. Microsurgical epididymal sperm aspiration and intracytoplasmic sperm injection: a new effective approach to infertility as a result of congenital bilateral absence of the vas deferens. Fertil Steril. 1994;61:1045–1051. 8194615 10.1016/s0015-0282(16)56754-5

[4] Devroey P, Liu J, Nagy Z, et al. Pregnancies after testicular sperm extraction and intracytoplasmic sperm injection in non-obstructive azoospermia. Hum Reprod. 1995;10:1457–1460. 7593514 10.1093/humrep/10.6.1457

[5] Talreja D, Gupta C, Pai H, et al. Comparative Analysis of Surgically Retrieved Sperms in Cases of Obstructive and Nonobstructive Azoospermia Treated for Infertility. J Hum Reprod Sci. 2020;13:201–208. 33311906 10.4103/jhrs.JHRS_41_19 PMC7727882

[6] Ohlander S, Hotaling J, Kirshenbaum E, et al. Impact of fresh versus cryopreserved testicular sperm upon intracytoplasmic sperm injection pregnancy outcomes in men with azoospermia due to spermatogenic dysfunction: a meta-analysis. Fertil Steril. 2014;101:344–349. 24345355 10.1016/j.fertnstert.2013.10.012

[7] Esteves SC, Roque M, Bradley CK, et al. Reproductive outcomes of testicular versus ejaculated sperm for intracytoplasmic sperm injection among men with high levels of DNA fragmentation in semen: systematic review and meta-analysis. Fertil Steril. 2017;108:456–467.e1. 28865546 10.1016/j.fertnstert.2017.06.018

[8] Ambar RF, Agarwal A, Majzoub A, et al. The Use of Testicular Sperm for Intracytoplasmic Sperm Injection in Patients with High Sperm DNA Damage: a Systematic Review. World J Mens Health. 2020. DOI:10.5534/wjmh.200084. PMC8255394 32648379

[9] Lepine S, McDowell S, Searle LM, et al. Advanced sperm selection techniques for assisted reproduction. Cochrane Database Syst Rev. 2019;7:CD010461. 31425620 10.1002/14651858.CD010461.pub3 PMC6699650

[10] Tournaye H. Update on surgical sperm recovery--the European view. Hum Fertil (Camb). 2010;13:242–246. 21117934 10.3109/14647273.2010.522677

[11] Ozkavukcu S, Ibis E, Kizil S, et al. A laboratory modification to testicular sperm preparation technique improves spermatogenic cell yield. Asian J Androl. 2014;16:852–857. 25038178 10.4103/1008-682X.132468 PMC4236328

[12] Muller CH, Pagel ER. Recovery, isolation, identification, and preparation of spermatozoa from human testis. Methods Mol Biol. 2013;927:227–240. 22992917 10.1007/978-1-62703-038-0_20

[13] Crabbé E, Verheyen G, Silber S, et al. Enzymatic digestion of testicular tissue may rescue the intracytoplasmic sperm injection cycle in some patients with non-obstructive azoospermia. Hum Reprod. 1998;13:2791–2796. 9804232 10.1093/humrep/13.10.2791

[14] Modarresi T, Sabbaghian M, Shahverdi A, et al. Enzymatic digestion improves testicular sperm retrieval in non-obstructive azoospermic patients. Iran J Reprod Med. 2013;11:447–452. 24639777 PMC3941318

[15] Baukloh V. Retrospective multicentre study on mechanical and enzymatic preparation of fresh and cryopreserved testicular biopsies. Hum Reprod. 2002;17:1788–1794. 12093841 10.1093/humrep/17.7.1788

[16] Nagy ZP, Verheyen G, Tournaye H, et al. An improved treatment procedure for testicular biopsy specimens offers more efficient sperm recovery: case series. Fertil Steril. 1997;68:376–379. 9240275 10.1016/s0015-0282(97)81534-8

[17] Wöber M, Ebner T, Steiner SL, et al. A new method to process testicular sperm: combining enzymatic digestion, accumulation of spermatozoa, and stimulation of motility. Arch Gynecol Obstet. 2015;291:689–694. 25216961 10.1007/s00404-014-3458-3

[18] Ebner T, Tews G, Mayer RB, et al. Pharmacological stimulation of sperm motility in frozen and thawed testicular sperm using the dimethylxanthine theophylline. Fertil Steril. 2011;96:1331–1336. 21962960 10.1016/j.fertnstert.2011.08.041

[19] Tournaye H, Wieme P, Janssens R, et al. Incubation of spermatozoa from asthenozoospermic semen samples with pentoxifylline and 2-deoxyadenosine: variability in hyperactivation and acrosome reaction rates. Hum Reprod. 1994;9:2038–2043. 7868671 10.1093/oxfordjournals.humrep.a138390

[20] Esteves SC, Spaine DM, Cedenho AP. Effects of pentoxifylline treatment before freezing on motility, viability and acrosome status of poor quality human spermatozoa cryopreserved by the liquid nitrogen vapor method. Brazilian J Med Biol Res = Rev Bras Pesqui Medicas E Biol. 2007;40:985–992. 10.1590/s0100-879x2006005000118 17653453

[21] Fountain S, Rizk B, Avery S, et al. An evaluation of the effect of pentoxifylline on sperm function and treatment outcome of male-factor infertility: a preliminary study. J Assist Reprod Genet. 1995;12:704–709. 8624427 10.1007/BF02212897

[22] Laokirkkiat P, Kunathikom S, Choavaratana R, et al. Comparison between sperm treated with pentoxifylline and 2-deoxyadenosine using hypo-osmotic swelling test. J Med Assoc Thai. 2007;90:211–215. 17375622

[23] Kovacic B, Vlaisavljevic V, Reljic M. Clinical use of pentoxifylline for activation of immotile testicular sperm before ICSI in patients with azoospermia. J Androl. 2006;27:45–52. 16400077 10.2164/jandrol.05079

[24] Griveau J-F, Lobel B, Laurent M-C, et al. Interest of pentoxifylline in ICSI with frozen-thawed testicular spermatozoa from patients with non-obstructive azoospermia. Reprod Biomed Online. 2006;12:14–18. 16454927 10.1016/s1472-6483(10)60974-1

[25] Terriou P, Hans E, Giorgetti C, et al. Pentoxifylline initiates motility in spontaneously immotile epididymal and testicular spermatozoa and allows normal fertilization, pregnancy, and birth after intracytoplasmic sperm injection. J Assist Reprod Genet. 2000;17:194–199. 10955242 10.1023/A:1009435732258 PMC3455468

[26] Tournaye H, Van Der Linden M, Van Den Abbeel E, et al. Effect of pentoxifylline on implantation and post-implantation development of mouse embryos in vitro. Hum Reprod. 1993;8:1948–1954. 8288765 10.1093/oxfordjournals.humrep.a137966

[27] Tournaye H, Van Der Linden M, Van Den Abbeel E, et al. Effects of pentoxifylline on in-vitro development of preimplantation mouse embryos. Hum Reprod. 1993;8:1475–1480. 8253939 10.1093/oxfordjournals.humrep.a138282

[28] Yovich JM, Edirisinghe WR, Yovich JL. Use of the acrosome reaction to ionophore challenge test in managing patients in an assisted reproduction program: a prospective, double-blind, randomized controlled study. Fertil Steril. 1994;61:902–910. 8174729 10.1016/s0015-0282(16)56704-1

[29] Ebner T, Köster M, Shebl O, et al. Application of a ready-to-use calcium ionophore increases rates of fertilization and pregnancy in severe male factor infertility. Fertil Steril. 2012;98:1432–1437. 22921909 10.1016/j.fertnstert.2012.07.1134

[30] Parinaud J, Vieitez G, Moutaffian H, et al. Relevance of acrosome function in the evaluation of semen in vitro fertilizing ability. Fertil Steril. 1995;63:598–603. 7851593 10.1016/s0015-0282(16)57432-9

[31] Liu DY, Baker HW. Relationship between the zona pellucida (ZP) and ionophore A23187-induced acrosome reaction and the ability of sperm to penetrate the ZP in men with normal sperm-ZP binding**Supported by grant no. 930628 from National Health and Medical Research Council, Canberra, Australian Capital Territory, Australia. Fertil Steril. 1996;66(2):312–315. 8690122 10.1016/s0015-0282(16)58459-3

[32] Hallak J, Cocuzza M, Sarkis AS, et al. Organ-sparing microsurgical resection of incidental testicular tumors plus microdissection for sperm extraction and cryopreservation in azoospermic patients: surgical aspects and technical refinements. Urology. 2009;73:882–887. 10.1016/j.urology.2008.08.510 19201456

[33] Casemiro Monteiro RA, Pariz JR, De Campos Pieri P, et al. An easy, reproducible and cost-effective method for andrologists to improve the laboratory diagnosis of nonobstructive azoospermia: a novel microcentrifugation technique. Int Braz J Urol. 2016;42:132–138. 27136479 10.1590/S1677-5538.IBJU.2015.0090 PMC4811238

[34] Hendin BN, Patel B, Levin HS, et al. Identification of spermatozoa and round spermatids in the ejaculates of men with spermatogenic failure. Urology. 1998;51:816–819. 9610597 10.1016/s0090-4295(98)00007-7

[35] Jeyendran RS, Van Der Ven HH, Perez-Pelaez M, et al. Development of an assay to assess the functional integrity of the human sperm membrane and its relationship to other semen characteristics. J Reprod Fertil. 1984;70:219–228. 6694140 10.1530/jrf.0.0700219

[36] Rossato M, Galeazzi C, Ferigo M, et al. Antisperm antibodies modify plasma membrane functional integrity and inhibit osmosensitive calcium influx in human sperm. Hum Reprod. 2004;19:1816–1820. 15205398 10.1093/humrep/deh317

[37] Soares JB, Glina S, Antunes NJ, et al. Sperm tail flexibility test: a simple test for selecting viable spermatozoa for intracytoplasmic sperm injection from semen samples without motile spermatozoa. Rev Hosp Clin Fac Med Sao Paulo. 2003;58:250–253. 14666321 10.1590/s0041-87812003000500003

[38] Oliveira NM, Vaca Sánchez R, Rodriguez Fiesta S, et al. Pregnancy with frozen-thawed and fresh testicular biopsy after motile and immotile sperm microinjection, using the mechanical touch technique to assess viability. Hum Reprod. 2004;19:262–265. 14747164 10.1093/humrep/deh083

[39] Charehjooy N, Najafi MH, Tavalaee M, et al. Selection of Sperm Based on Hypo-Osmotic Swelling May Improve ICSI Outcome: a Preliminary Prospective Clinical Trial. Int J Fertil Steril. 2014;8:21–28. 24695913 PMC3973167

[40] Sallam HN, Farrag A, Agameya A-F, et al. The use of the modified hypo-osmotic swelling test for the selection of immotile testicular spermatozoa in patients treated with ICSI: a randomized controlled study. Hum Reprod. 2005;20:3435–3440. 16126756 10.1093/humrep/dei249

[41] Hauser R, Yavetz H, Paz GF, et al. The predictive fertilization value of the hypoosmotic swelling test (HOST) for fresh and cryopreserved sperm. J Assist Reprod Genet. 1992;9:265–270. 1525459 10.1007/BF01203826

[42] Check JH, Katsoff B, Yuan W, et al. Intracytoplasmic sperm injection completely negates the implantation problem associated with conventional fertilization with sperm with low hypo-osmotic swelling test scores as evidenced by evaluating donor-recipient pairs. Clin Exp Obstet Gynecol. 2012;39:21–22. 22675948

[43] El-Nour AM, Al Mayman HA, Jaroudi KA, et al. Effects of the hypo-osmotic swelling test on the outcome of intracytoplasmic sperm injection for patients with only nonmotile spermatozoa available for injection: a prospective randomized trial. Fertil Steril. 2001;75:480–484. 11239527 10.1016/s0015-0282(00)01762-3

[44] Mangoli V, Mangoli R, Dandekar S, et al. Selection of viable spermatozoa from testicular biopsies: a comparative study between pentoxifylline and hypoosmotic swelling test. Fertil Steril. 2011;95:631–634. 21074155 10.1016/j.fertnstert.2010.10.007

[45] Aktan TM, Montag M, Duman S, et al. Use of a laser to detect viable but immotile spermatozoa. Andrologia. 2004;36:366–369. 15541052 10.1111/j.1439-0272.2004.00636.x

[46] Nordhoff V, Schüring AN, Krallmann C, et al. Optimizing TESE-ICSI by laser-assisted selection of immotile spermatozoa and polarization microscopy for selection of oocytes. Andrology. 2013;1:67–74. 23258632 10.1111/j.2047-2927.2012.00020.x

[47] Riva NS, Ruhlmann C, Iaizzo RS, et al. Comparative analysis between slow freezing and ultra-rapid freezing for human sperm cryopreservation. JBRA Assist Reprod. 2018;22:331–337. 30132630 10.5935/1518-0557.20180060 PMC6210620

[48] Schachter-Safrai N, Karavani G, Levitas E, et al. Does cryopreservation of sperm affect fertilization in nonobstructive azoospermia or cryptozoospermia? Fertil Steril. 2017;107:1148–1152. 28392002 10.1016/j.fertnstert.2017.03.009

[49] Isachenko V, Isachenko E, Katkov II, et al. Cryoprotectant-free cryopreservation of human spermatozoa by vitrification and freezing in vapor: effect on motility, DNA integrity, and fertilization ability. Biol Reprod. 2004;71:1167–1173. 15175233 10.1095/biolreprod.104.028811

[50] Le MT, Nguyen TTT, Nguyen TT, et al. Cryopreservation of human spermatozoa by vitrification versus conventional rapid freezing: effects on motility, viability, morphology and cellular defects. Eur J Obstet Gynecol Reprod Biol. 2019;234:14–20. 30640121 10.1016/j.ejogrb.2019.01.001

[51] Sherman JK. Synopsis of the use of frozen human semen since 1964: state of the art of human semen banking. Fertil Steril. 1973;24:397–412. 4735423 10.1016/s0015-0282(16)39678-9

[52] Sharma Y, Sperm Cryopreservation: SM. Principles and Biology. J Infertil Reprod Biol. 2020;8:43–48.

[53] Esteves SC, Miyaoka R, Agarwal A. Sperm retrieval techniques for assisted reproduction. Int Braz J Urol. 2011;37:570–583. 22099268 10.1590/s1677-55382011000500002

[54] Lopes LS, Cury VN, Cha JD, et al. Do assisted reproduction outcomes differ according to aetiology of obstructive azoospermia? Andrologia. 2020;52:e13425. 31691344 10.1111/and.13425

[55] Corona G, Minhas S, Giwercman A, et al. Sperm recovery and ICSI outcomes in men with non-obstructive azoospermia: a systematic review and meta-analysis. Hum Reprod Update. 2019;25:733–757. 31665451 10.1093/humupd/dmz028

[56] Schlegel PN. Testicular sperm extraction: microdissection improves sperm yield with minimal tissue excision. Hum Reprod. 1999;14:131–135. 10374109 10.1093/humrep/14.1.131

[57] Flannigan RK, Schlegel PN. Microdissection testicular sperm extraction: preoperative patient optimization, surgical technique, and tissue processing. Fertil Steril. 2019;111:420–426. 30827516 10.1016/j.fertnstert.2019.01.003

[58] Popal W, Nagy ZP. Laboratory processing and intracytoplasmic sperm injection using epididymal and testicular spermatozoa: what can be done to improve outcomes? Clinics (Sao Paulo). 2013;68 Suppl 1:125–130. 23503962 10.6061/clinics/2013(Sup01)14 PMC3583163

[59] Kaur KK, Allahbadia G, Singh M. Management of azoospermia with special emphasis on microdissection testicular sperm extraction (mTESE) in non obstructive azoospermia prior to IVF/ICSI to optimize sperm retrieval rates, pregnancy, live birth rates – a systematic review. Syst Biol Reprod Med. 2016;62 (6): 359-371. doi:10.15406/mojs.2020.08.00162

[60] Verheyen G, Popovic-Todorovic B, Tournaye H. Processing and selection of surgically-retrieved sperm for ICSI: a review. Basic Clin Androl. 2017;27:6. 28331619 10.1186/s12610-017-0050-2 PMC5360083

[61] WHO. Examination and processing of human semen. World Heal Organ. 2010;286. Edition, F.

[62] Simopoulou M, Gkoles L, Bakas P, et al. Improving ICSI: a review from the spermatozoon perspective. Syst Biol Reprod Med. 2016;62:359–371. 27646677 10.1080/19396368.2016.1229365

[63] Nordhoff V. How to select immotile but viable spermatozoa on the day of intracytoplasmic sperm injection? An embryologist’s view. Andrology. 2015;3:156–162. 25331054 10.1111/andr.286

[64] Liu S, Li F. Cryopreservation of single-sperm: where are we today? Reprod Biol Endocrinol. 2020;18:41. 32398019 10.1186/s12958-020-00607-x PMC7216378

[65] Armenian SH, Landier W, Hudson MM, et al. Children’s Oncology Group’s 2013 blueprint for research: survivorship and outcomes. Pediatr Blood Cancer. 2013;60:1063–1068. 23255494 10.1002/pbc.24422 PMC3799776

[66] Corkum KS, Lautz TB, Johnson EK, et al. Testicular wedge biopsy for fertility preservation in children at significant risk for azoospermia after gonadotoxic therapy. J Pediatr Surg. 2019;54:1901–1905. 30853246 10.1016/j.jpedsurg.2019.01.055

[67] Lautz TB, Harris CJ, Laronda MM, et al. A fertility preservation toolkit for pediatric surgeons caring for children with cancer. Semin Pediatr Surg. 2019;28:150861. 31931969 10.1016/j.sempedsurg.2019.150861

[68] Berkovitz A, Miller N, Silberman M, et al. A novel solution for freezing small numbers of spermatozoa using a sperm vitrification device. Hum Reprod. 2018;33:1975–1983. 30285105 10.1093/humrep/dey304

